# Experimental validation of multi-fraction online adaptations in magnetic resonance guided radiotherapy

**DOI:** 10.1016/j.phro.2023.100507

**Published:** 2023-11-09

**Authors:** Madelon van den Dobbelsteen, Sara L. Hackett, Bram van Asselen, Stijn Oolbekkink, Jochem W.H. Wolthaus, J.H. Wilfred de Vries, Bas W. Raaymakers

**Affiliations:** a Department of Radiotherapy, University Medical Center Utrecht, Heidelberglaan 100, 3584 CX Utrecht, The Netherlands

**Keywords:** MR-linac, Online adaptation, Quality assurance, Multi-fraction treatment

## Abstract

**Background and purpose:**

Radiotherapy plan verification is generally performed on the reference plan based on the pre-treatment anatomy. However, the introduction of online adaptive treatments demands a new approach, as plans are created daily on different anatomies. The aim of this study was to experimentally validate the accuracy of total doses of multi-fraction plan adaptations in magnetic resonance imaging guided radiotherapy in a phantom study, isolated from the uncertainty of deformable image registration.

**Materials and methods:**

We experimentally verified the total dose, measured on external beam therapy 3 (EBT3) film, using a treatment with five online adapted fractions. Three series of experiments were performed, each focusing on a category of inter-fractional variation; translations, rotations and body modifications. Variations were introduced during each fraction and adapted plans were generated and irradiated. Single fraction doses and total doses over five online adapted fractions were investigated.

**Results:**

The online adapted measurements and calculations showed a good agreement for single fractions and multi-fraction treatments for the dose profiles, gamma passing rates, dose deviations and distances to agreement. The gamma passing rate using a 2%/2 mm criterion ranged from 99.2% to 99.5% for a threshold dose of 10% of the maximum dose (D_*max*_) and from 96.2% to 100% for a threshold dose of 90% of D_*max*_, for the total translations, rotations and body modifications.

**Conclusions:**

The total doses of multi-fraction treatments showed similar accuracies compared to single fraction treatments, indicating an accurate dosimetric outcome of a multi-fraction treatment in adaptive magnetic resonance imaging guided radiotherapy.

## Highlights

• Experimentally validated high accuracy of multi-fraction online adapted plans.
• Accuracy of total dose isolated from uncertainty of deformable image registration.
• Multi-fraction treatments show similar accuracies as single fraction treatments.

## 1 Introduction

Online adaptive radiation therapy gives the opportunity to account for daily anatomical changes and research is ongoing due to evolving technologies and clinical trials [1]. Treatment plans are adapted based on images acquired during each fraction. These images expose information that enables better targeting of the tumor and reduce healthy tissue exposure [2], [3], [4]. During an online adaptive radiotherapy treatment, the treatment plan is optimized and recalculated using the planning constraints and daily patient contours [2].

Patient plan verification is generally performed on the reference plan based on the pre-treatment anatomy [5]. However, the introduction of online adaptive treatments demands a new approach, as plans are created daily on different anatomies [1], [6]. Previous studies focused on experimental verification of single fraction online adaptive plans [7], [8], [9]. To obtain the overall accuracy of a multi-fraction treatment, quality assurance (QA) should be performed on the total dose. However, the entire online adaptive treatment consists of different online adapted plans calculated on different anatomies. Therefore, patient-specific QA is often performed on individual pre-treatment or online adapted fractions instead of the total series of online adaptive fractions [5]. Deformable image registration (DIR) has been used to accumulate the dose of online adapted plans using dose warping [10], [11], [12], however this approach introduces an uncertainty in the accumulated dose and difficulties verifying correctness of DIR [13], [14], [15].

We experimentally verified the accuracy of single fraction doses and total dose, measured on external beam therapy 3 (EBT3) film, using a treatment with five online adapted fractions on the Magnetic Resonance Imaging Linear Accelerator (MRI-Linac) using ICRU-97 terminology [16], in this research denoted as MR-linac. The aim of this study was to experimentally validate the accuracy of total doses of multi-fraction plan adaptations without introducing the uncertainty of DIR.

## 2 Materials and methods

On the 1.5T Unity MR-linac (Elekta AB, Sweden), plans can be adapted via the Adapt-To-Shape (ATS) method [2]. A five fraction MR-linac treatment was planned and delivered, using different inter-fractional variations of the phantom. Measured and calculated doses were compared for single fractions and total treatments with multiple fractions, using the QUASAR MRI^*4D*^ motion phantom (IBA QUASAR, Modus Medical Devices Inc., Canada).

### 2.1 Inter-fractional variations

**Fig. 1:**
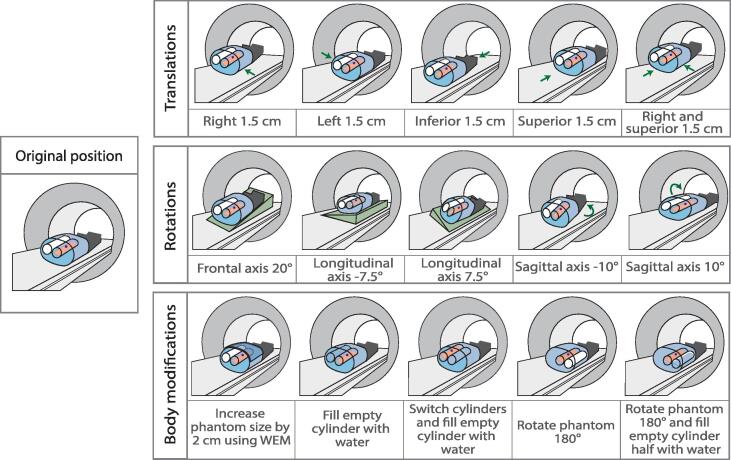
Inter-fractional variations. Original position of the phantom and three categories of positional and anatomical inter-fraction variations of the phantom; translations, rotations and body modifications. The red plane represents the film, the blue dot represents the ball bearing and the green region represents the ramp. The phantom contains water (blue) or air (white). Abbreviation: Water Equivalent Material (WEM).

Three series of experiments were performed, each focusing on a category of inter-fractional variation; translations, rotations and body modifications. The variations of phantom position or form were introduced at the start of each fraction. Measurements were performed using the QUASAR phantom, comprising an external body and two internal cylinders. One cylinder contained a holder for a measurement device, for relative dosimetry (film) or absolute dosimetry (ionization chamber), and the other (empty) cylinder represents the lung. To avoid air gaps which influence the dosimetric outcome, water was added around the film and the film cassette [17]. A schematic view of the phantom and the inter-fractional variations including precise adaptation dimensions is given in Fig. 1. More details on the inter-fractional variations are enclosed in the supplementary material (A). The total dose distribution comprised summed doses of five fractions. For single fraction measurements, the same inter-fractional variation was used for the corresponding fraction in the total dose measurements. For the first and last fraction of the rotations and body modifications, no additional plan was made and the total plan matching the fraction was reused. In total fourteen datasets were acquired, three total doses and eleven single fractions. The single fractions were measured and calculated for the following experiments to cover the full range of inter-fractional variations: the original plan without adaptation, all five translation fractions, rotation fraction 1, 3 and 5, and body modifications fraction 1 and 5.

### 2.2 Plan setup and adaptation

The original treatment plan comprised thirteen beams, using a scheme of five fractions of 1.2 Gy based on a rescaled clinical template for Stereotactic Body Radiation Therapy for a tumor in the right lung. All plans received a minimum coverage of 97.9% of the planning target volume with a total prescription dose of 6.0 Gy. In the calculated reference plan the maximum dose (D_*max*_) was 1.7 Gy for the single fraction and 8.6 Gy for the total five fraction treatment. The D_*max*_ per fraction was chosen to fit the range of Gafchromic EBT3 film (Ashland Inc., USA)(LOT 01042103) for single and multi-fraction doses. Beam angles were chosen to minimize dose to the left lung. A schematic view of the original plan, made in the treatment planning system Monaco (Elekta AB, Sweden)(v5.51.10), is illustrated in the supplementary material (B). After each inter-fractional variation an MR-scan of the phantom was made. Contours were transferred from the original CT scan to the new scan using rigid translations in Monaco, and checked and edited if necessary. Bulk Electron densities (ED) were assigned to structures, based on average CT values. The daily MRI, including manually adapted structures with assigned EDs, were used for plan adaptation. A new plan was generated and calculated using the ATS method, using optimize weights and shapes from fluence [2].

### 2.3 Dose measurements and calculations

Prior to the measurements, a calibration curve of twenty data points was made for the used film batch, whereby film pieces were exposed to known doses to match the film response and the delivered dose in reference conditions [18]. Additionally, daily correction films were obtained whereby film pieces were again exposed to known doses. These daily correction films were scanned together with the final delivered film doses, whereby the calibration curve was scaled based on three to five daily correction films. The scaled calibration curve was used to convert the film response to delivered dose using an in-house software tool. For the total dose of the films, the daily calibration films ranged from 0 to 10 Gy, with four to five calibration doses in total. For the single fraction films, the daily calibration ranged from 0 to 2, 2.2 or 2.5 Gy, depending on the dataset, with three or four calibration doses in total.

For the ionization chamber measurements, the single fraction original plan and five single translation fractions were measured in the high dose region, using the Semiflex 0.125 cm^3^ ionization chamber (PTW-Freiburg, Germany)(TW31010 SN007898). Charge was converted to dose according to an existing calibration factor and a correction for temperature and pressure. The film insert was replaced with an insert for the ionization chamber.

Calculations were performed using the GPU-oriented Monte Carlo dose calculation algorithm (GPUMCD) in Monaco. For online plan adaptation a statistical uncertainty of 3% per control point and a grid size of 3 mm was used, but plans were recalculated for comparison with measurements using a statistical uncertainty of 0.3% per control point and a grid size of 2 mm.

### 2.4 Total dose and dose processing

**Fig. 2:**
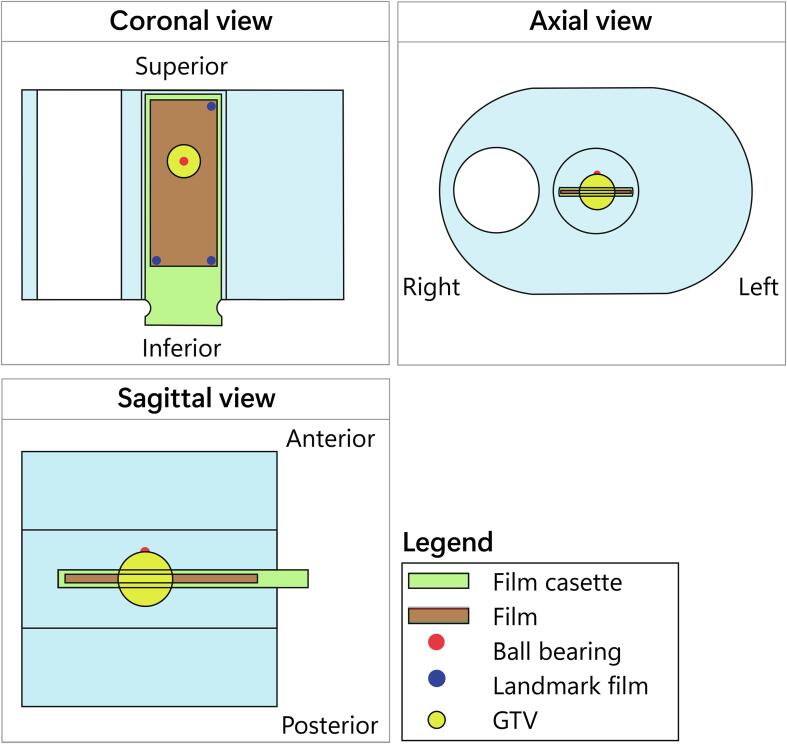
Schematic view phantom. Overview of the film cassette, film, ball bearing, film landmarks and gross tumor volume (GTV) in the coronal, axial and sagittal view.

To acquire the total measured dose over multi-fraction plan adaptations, the film cassette with EBT3 film remained in the cylinder during the delivery of all five fractions. Three pins on the edges of the cassette were used to hold the film in place, leading to three landmarks on the film. The landmark positions were manually selected using Matlab (Mathworks, USA)(version 2019a). These positions were used for rotational scanner corrections and to select the 2D dose grid. An overview of the landmarks on the film is illustrated in Fig. 2. Each plan adaptation was based on the positions of the inter-fractional variations of the phantom. Therefore, the calculated 3D dose grids including matching MR images and structure sets were available for both single fractions and the individual five fractions of the total dose. In order to compare the calculated dose to the measured film dose, the 2D dose grid the size of the film was selected out of this 3D dose grid in a processing step. More details are enclosed in the supplementary material (C). Doses of the five individual fractions were added to determine the total dose of the treatment. For ionization chamber measurements, the calculated dose was analyzed using a mean dose of a sphere with a radius of 0.3 cm at the position of the ionization chamber, based on the active volume of the ionization chamber.

Measurements and calculations for single and multi-fraction treatments were compared on the same 2D dose grid, whereby the original grids were re-sampled to a common grid size of 1x1 mm^2^, using interpolation. Relative calculated and measured dose distributions were compared. To obtain the relative dose, the dose was divided by the median dose in the center of the high dose region within a 10 mm radius. Between measured and calculated 2D grids, small displacements of up to 1.5 mm in crossline and inline directions were corrected for to remove setup and post processing errors. The film size is defined as 60x165 mm by the manufacturer. The defined post processing grid was 61x166 mm, but edge voxels were deleted to remove edge effects, leading to a final grid size of 59x164 mm. Dose differences in dose profiles were expressed in percentage point (pp) to avoid focusing on large relative differences in the low dose region. A global γ analysis of 2%/2 mm was performed, using the function CalcGamma in Matlab [19] and dose deviations were determined, both using threshold doses of 10% and 90% of D_*max*_. The dose deviations represented the voxel-wise dose difference between measurements and calculations without distance to agreement (DTA) correction. The DTA with a threshold dose of 10% - 90% of D_*max*_ was calculated (mean ± standard deviation (SD)). The smallest DTA was determined within 0.5% local dose deviation between measurements and calculations. For ionization chamber measurements, differences were calculated as the difference between measured and calculated doses, expressed as a percentage of the calculated dose.

## 3 Results

### 3.1 Relative dose differences

A comparison between measurements and calculations without normalization showed dose differences of 0.0% ± 2.6% (mean ± SD), as illustrated in the supplementary material (D). Displacements of 0.2 mm ± 0.7 mm and −0.3 mm ± 0.3 mm (mean ± SD) were shown for inline and crossline directions, respectively, as illustrated in the supplementary material (E).

**Fig. 3:**
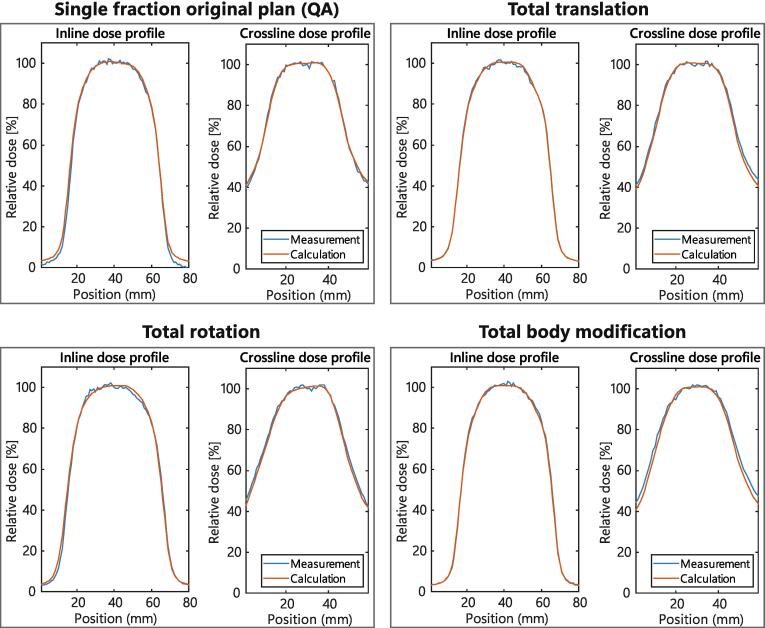
Inline and crossline dose profiles. Inline and crossline dose profiles for the single fraction original plan and the total translations, rotations, and body modifications. Perpendicular dose profiles intersecting the center of the high dose region dose are shown.

Fig. 3 illustrates dose profiles of measurements and calculations for four different datasets; the single fraction of the original plan and total doses of translations, rotations and body modifications. Mean dose differences, calculations subtracted from measurements, were −1.6 pp, 0.1 pp, −0.6 pp and 0.3 pp for the inline dose profiles, and −0.1 pp, 1.2 pp, 1.2 pp and 2.0 pp for the crossline dose profiles, for all four cases, respectively. For the single fraction original plan, larger dose differences were shown in the penumbra and low dose region of the inline profiles, with maximum dose differences of −7.2 pp. For total translation and total body modification dose, crossline profiles showed larger dose differences in the penumbra with maximum dose differences of 3.5 pp and 5.1 pp, respectively.

**Fig. 4:**
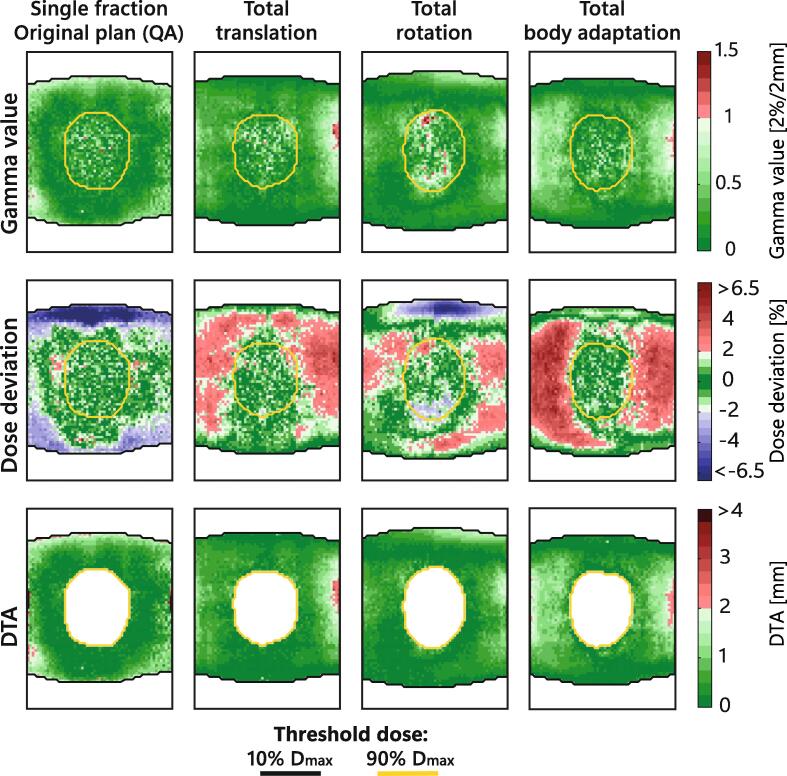
Gamma value, dose deviation and DTA. The γ value, dose deviation and distance to agreement (DTA), for the single fraction original plan and the total translations, rotations and body modifications. Threshold isodose lines of 10% and 90% of D_*max*_ are illustrated. The maximum dose deviation was between −6.5% and 6.5% for the total translations, rotations and body modifications. The single fraction of the original plan showed a region with dose deviations up to −10% and a single voxel outlier of 11.6%. The maximum DTA was between −4.0 mm and 4.0 mm for the total translations, rotations and body modifications. The single fraction of the original plan showed a maximum DTA of 6.8 mm.

Fig. 4 illustrates the γ values, the dose deviation and the DTA. The γ passing rates were 99.7%, 99.2%, 99.2% and 99.5% for a threshold dose of 10% of D_*max*_, and 99.5%, 99.6%, 96.2% and 100% for a threshold dose of 90% of D_*max*_, for the four cases, respectively. The dose deviations showed larger differences in the penumbra and low dose region, in concurrence with the dose profiles. Mean dose deviations were −1.1%, 1.5%, 0.5% and 1.7% for a threshold dose of 10% of D_*max*_ and 0.1%, 0.3%, 0.0% and 0.1% for a threshold dose of 90% of D_*max*_, for the four cases, respectively. The DTA values were low, with mean DTA values of 0.6 mm, 0.5 mm, 0.4 mm, and 0.7 mm, for the four cases, respectively.

Table 1 shows the γ passing rate, dose deviation and DTA for various threshold doses for all fourteen datasets. The γ passing rates ranged from 94.5% to 99.9% for 10% threshold dose and from 86.0% to 100% for 90% threshold dose. The third single fraction of the translation showed an unusually low γ passing rate of 86.0% for a threshold dose of 90%, whereas the γ passing rate for all other datasets was at least 96.2%. The total doses showed outcomes in the same range as the single fractions for the γ passing rates, the dose deviations and DTAs.

**Table 1:** The γ passing rate, dose deviation and distance to agreement (DTA) for various threshold doses for all fourteen available datasets.

		γ**passing rate** **2%/2** **mm [%]**		**Dose deviation [%]** **Mean**±**SD**		**DTA [mm]** **Mean**±**SD**
	*Threshold dose*	*10%*	*90%*	*10%*	*90%*	*10% - 90%*
Original plan	Single fraction	99.7	99.5	-1.1 ± 1.6	0.1 ± 0.8	0.6 ± 0.6
Translation	Total dose	99.2	99.6	1.5 ± 1.2	0.3 ± 0.9	0.5 ± 0.4
	Fraction 1	99.8	99.5	0.0 ± 1.3	0.2 ± 0.9	0.3 ± 0.4
	Fraction 2	99.8	99.2	0.4 ± 1.2	0.4 ± 0.9	0.3 ± 0.3
	Fraction 3	94.5	86.0	0.1 ± 1.5	0.6 ± 1.7	0.5 ± 1.4
	Fraction 4	99.7	98.9	0.1 ± 1.2	0.1 ± 0.9	0.2 ± 0.2
	Fraction 5	99.5	98.5	-0.5 ± 1.3	0.1 ± 1.0	0.3 ± 0.5
Rotation	Total dose	99.2	96.2	0.5 ± 1.7	0.0 ± 1.4	0.4 ± 0.4
	Fraction 1	99.8	99.4	0.1 ± 1.2	-0.4 ± 1.1	0.2 ± 0.3
	Fraction 3	99.6	98.4	-0.1 ± 1.1	0.2 ± 1.0	0.2 ± 0.3
	Fraction 5	99.9	99.6	-0.9 ± 1.8	0.1 ± 0.9	0.4 ± 0.5
Body modification	Total dose	99.5	100	1.7 ± 1.7	0.1 ± 0.9	0.7 ± 0.5
	Fraction 1	99.0	99.5	-0.7 ± 1.5	0.3 ± 0.8	0.4 ± 1.0
	Fraction 5	97.2	98.8	-1.8 ± 1.5	-0.1 ± 0.9	0.8 ± 1.0

### 3.2 Absolute dose differences

The ionization chamber doses ranged from 1.7 Gy to 1.8 Gy, for all datasets. Dose differences between measurements and calculations were −1.9% for the original plan, and the dose difference of the single fraction translations ranged from 1.2% to 1.7%.

## 4 Discussion

In this study we experimentally validated the accuracy of total doses of multi-fraction plan adaptations on the MR-linac without introducing the uncertainty of DIR. Excellent agreement was found between total measured and calculated dose distributions, based on relative dose comparisons for inter-fractional variations (translations, rotations and body modifications). This excellent agreement was shown for dose profiles, γ passing rates, dose deviations and DTAs. The total doses of multi-fraction treatments show similar accuracies compared to single fraction treatments, indicating highly accurate dosimetry of multi-fraction online adaptive treatment using the ATS workflow on the MR-linac, if uncertainties associated with DIR can be controlled.

GPUMCD provides reliable dose calculations with a low uncertainty for single fields [20]. Multiple measurement devices can be used in combination with this phantom. In this research a film was used to ensure high spatial resolution, supplemented with single point measurements. Alternatively, multiple point measurements could be performed [10] or 3D gel measurements with a higher uncertainty could be performed [9], [21]. In general, Gafchromic EBT3 films have a low combined dosimetric uncertainty if factors influencing the film response can be sufficiently controlled [22], [23], [24]. However, film artefacts, the strong magnetic field effects (shown in EBT2 films) and the scan-to-scan variability can increase the dosimetric uncertainty [18], [25], [26]. As the experiments took several hours, the irradiation-to-scanning time periods differed per film, which increases the uncertainty in the film readout [27]. Our dosimetric differences between Gafchromic film and calculations ranged from −4.0% to 5.3%. Hence, we have used both absolute and relative distributions, and absolute differences between calculated doses and Semiflex chamber measurements were much smaller, ranging from −1.9% to 1.7%. An end-to-end test of the MR-linac workflow using Gafchromic film showed subvoxel uncertainties between 0.1 mm and 0.9 mm [7]. In our research displacements were slightly higher, with inline and crossline displacements ranging from −0.6 mm to 1.5 mm and −1.1 mm to 0.2 mm, respectively, but our study was not designed to find subvoxel uncertainty. In the post processing steps, the determination of the film cassette position and rotation were rounded, due to a fixed voxel size of 1 mm. Additionally, the manual selection of landmarks by the user and the positioning of the ball bearing could lead to geometrical uncertainties.

MR guided radiotherapy gives the opportunity to apply online adaptations based on the images acquired during each fraction [1], [6]. In this research we focus on the ATS procedure from Elekta. However, adaptation methods such as the adapt-to-position method on the Unity [2] and other MR-guided machines are available, such as the MRIdian [28]. Other institutes performed similar research, focussing on single rather than multi-fraction MR-guided treatments, illustrating experimental validation of online adaptive treatments using EBT3 films on a (deformable) phantom [7], [8], [9]. For an online adaptive treatment a γ passing rate of 93.1% was shown using absolute dosimetry and a threshold dose of 20% of D_*max*_ [9]. Here, the γ passing rate ranged from 94.5% to 99.8% using relative dosimetry and a threshold dose 10% of D_*max*_, indicating highly accurate adaptive doses. The third single fraction of the translations showed an unusually low γ passing rate compared to the other measurements, with 94.5% and 86.0% γ passing rates for a threshold dose of 10% and 90% of D_*max*_, respectively. However, the shape, location, and average dose of the high dose area show good agreement between measurements and calculations. Inhomogeneities in the film or conversion of the dose may have led to lower γ passing rates in this fraction [26]. Mittauer et al. investigated DIR and multi-fraction dose accumulation accuracy, using thermoluminescent dosimeters [10]. Other studies used patient data to accumulate the dose over multiple fractions using DIR [12], [29]. Bohoudi et al. illustrated two accumulated dose approaches in a deformable phantom [11]. The contour-based DIR approach showed an average dose deviation from accumulated film measurements for rectum and bladder surfaces of 0.6% and 0.3%, respectively. Compared to these previous studies, this is the only study experimentally verifying the total dose, without the uncertainty of DIR. Both this research and previous studies illustrated good dose accuracy for online adaptive single fractions. However, since every fraction is delivered on different anatomies, using different treatment plans, it is important to accurately determine the total delivered dose over multiple fractions.

With the introduction of the MR-linac, it is feasible to adapt the treatment plans based on the daily anatomy. This study indicates accurate ATS procedures for multi-fraction treatments. The possibility of real-time imaging gives the opportunity for intra-fraction adaptations [30], which opens the door to dose guided optimisation. The delivered dose could be determined using real-time methods to determine the dose, such as Electronic Portal Imaging Device and logfiles [31], [32]. The delivered dose should be accumulated for these intra-fraction adaptations on changing anatomies, probably using DIR [33].

We quantified the uncertainty associated with measurements of single and multiple online-adapted treatments, which is a necessary step towards quantifying the uncertainty associated with online adaptive treatments including DIR. The total doses of multi-fraction treatments show similar accuracies compared to single fraction treatments, indicating an accurate dosimetric outcome of a multi-fraction treatment using ATS adaptations on the MR-linac.

## Declaration of Competing Interest

The authors declare the following financial interests/personal relationships which may be considered as potential competing interests: The authors acknowledge funding by the Dutch Research Council (NWO) through project No. 18495 (ADEQUATE).
