# Supplementary material for: Experimental validation of multi-fraction online adaptations in magnetic resonance guided radiotherapy
Source: Phys Imaging Radiat Oncol. 2023 Nov 9;28:100507. doi: 10.1016/j.phro.2023.100507 (PMC10685304; doi:10.1016/j.phro.2023.100507)
Supplement: Supplementary data 1 [file mmc1.pdf]

Supplementary Material: Experimental validation of multi-fraction online adaptations in magnetic resonance guided radiotherapy

Madelon van den Dobbelsteen, Sara L. Hackett, Bram van Asselen, Stijn Oolbekkink, Jochem W.H. Wolthaus, J.H. Wilfred de Vries, Bas W. Raaymakers

A. Details inter-fractional variations

For the five translation fractions, the phantom was rigidly translated by 1.5 cm in one or more directions in the same plane. These translations represent typical setup variations for patients. The rotations and body adaptations were performed to test the adaptation method and summation for extreme situations. The phantom was rotated about a single axis only, using ramps orientated along the frontal, longitudinal or sagittal axis. The inter-fractional variations were extreme for the body modifications, but plan adaptations were controllable as each fraction examined the modification of a single structure or a translation of internal structures. For the body modifications, the phantom was adjusted by increasing the phantom size with Water Equivalent Material (WEM), or by changing the structure or density of components of the phantom.

B. Schematic view treatment plan

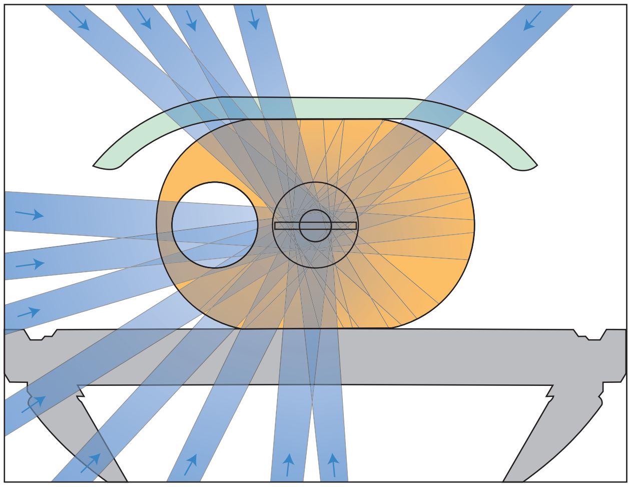

Figure B.1: Schematic view treatment plan. Schematic view of the phantom including generated beams in the treatment plan.

C. Defining 2D dose grid

Using a combination of the position of the ball bearing, selected rotations of the film cassette and the fixed grid size of the film, a 2D dose grid was defined. The ball bearing, positioned at the edge of the gross tumour volume (GTV), acted as a landmark position which was clearly visualized on the MR-scans, and had a fixed position above the film cassette. Using Matlab, the edges of the cylinder were automatically selected on three slices, after manually cropping the cylinder region. The mean positions of the cylinder on three slices were used to determine the angle around the frontal axis. The middle slice was selected, perpendicular to the frontal axis and the ball bearing. On five slices (including the middle slice), the edges of the cylinder were automatically selected, after manually cropping the cylinder region. The edges of the film cassette were selected on four points on the upper region of the cassette and four points at the lower region of the cassette on the middle MR slice. This led to detected rotations of the film cassette around all axes.

D. Difference normalization dose

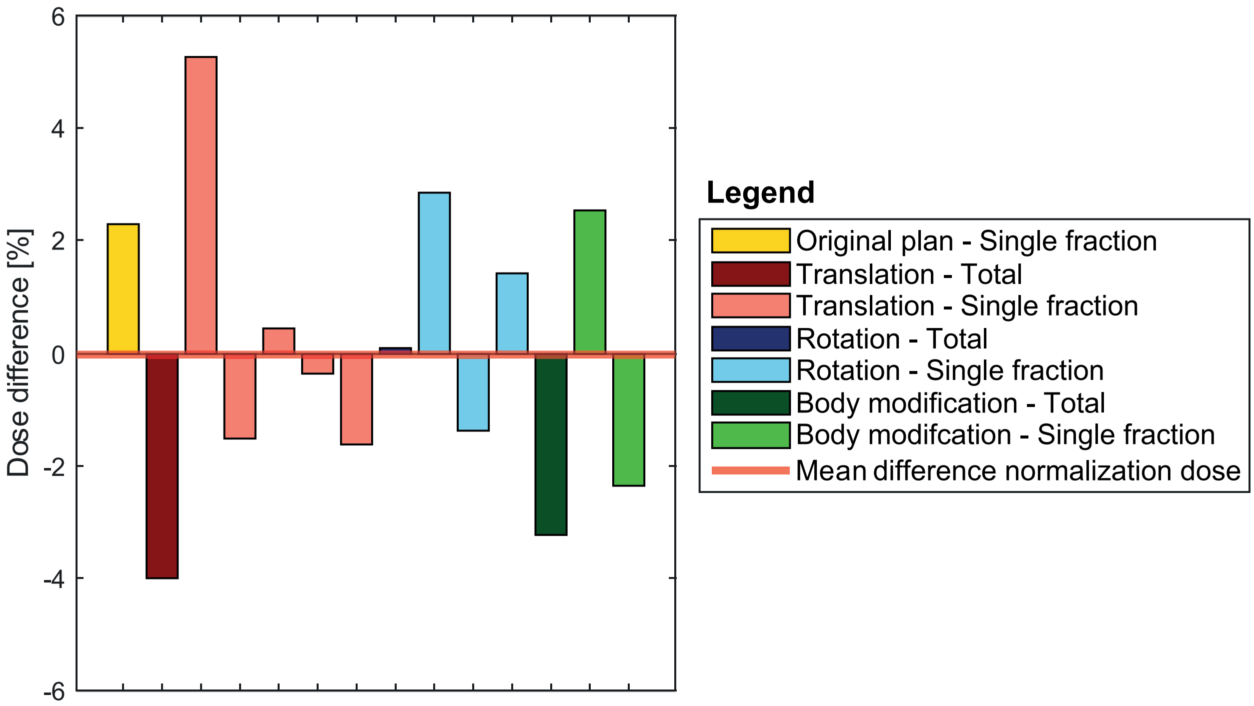

Figure D.1: Difference normalization dose for all fourteen datasets. The normalization dose is the median dose of a circle in the center of the high dose region of the 2D dose grid. The dose differences were calculated as the difference between the measured and calculated value, expressed as a percentage of the calculated value. The single fractions were measured and calculated for the following inter-fractional variations: all five translation fractions, rotations fraction 1,3 and 5, and body modifications fraction 1 and fraction 5.

E. Displacements

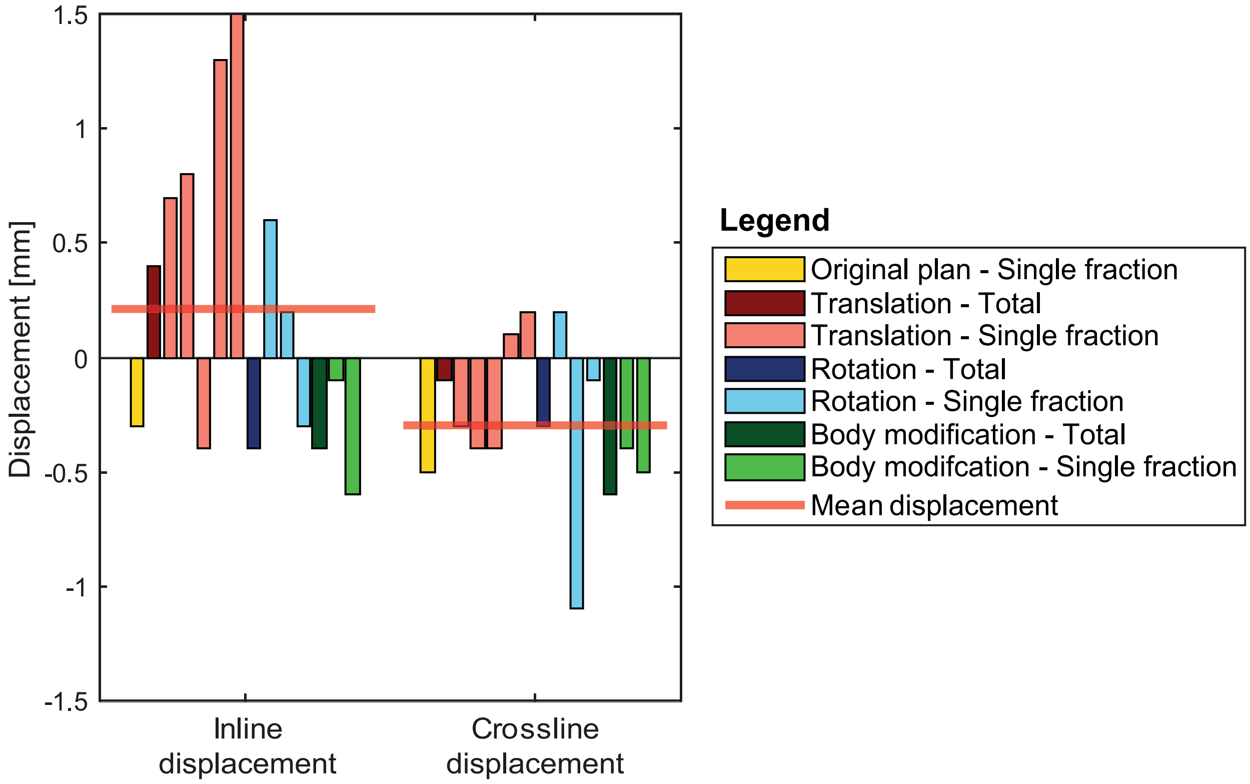

Figure E.1: Displacements. Displacements of the calculated dose to the measured dose in inline and crossline direction. The single fractions were measured and calculated for the following inter-fractional variations: all five translation fractions, rotations fraction 1,3 and 5, and body modifications fraction 1 and fraction 5.
